# Stress Response of Beagle Dogs to Repeated Short-Distance Road Transport

**DOI:** 10.3390/ani10112114

**Published:** 2020-11-14

**Authors:** Johannes Herbel, Jörg Aurich, Camille Gautier, Maria Melchert, Christine Aurich

**Affiliations:** 1 Obstetrics and Reproduction, Department for Small Animals and Horses, Vetmeduni Vienna, 1210 Vienna, Austria; johannes.herbel@vetmeduni.ac.at (J.H.); joerg.aurich@vetmeduni.ac.at (J.A.); 2 Artificial Insemination and Embryo Transfer, Department for Small Animals and Horses, Vetmeduni Vienna, 1210 Vienna, Austria; camillemarie.gautier@vetmeduni.ac.at (C.G.); maria.melchert@vetmeduni.ac.at (M.M.)

**Keywords:** dog, transport, stress, motions sickness, cortisol, heart rate, behavior

## Abstract

**Simple Summary:**

In our study, we investigated how Beagle dogs that had not been transported before respond to transport by car. The stress-associated hormone cortisol, heart rate, white blood cells and the dogs’ behavior were analyzed. We hypothesized that transport is perceived as a stressor but dogs get used to it with repeated transports. The dogs were studied in their kennels, in a transport cage placed in a stationary vehicle and during repeated one and two-hour transports. Cortisol concentration increased during transports, and this increase was also evident when dogs were transported repeatedly. Additionally, changes in white blood cells indicated a stress response. Heart rate increased at the beginning of transports, independent from the dogs’ transport experience. During transports but also in the stationary vehicle, dogs were mostly sitting, and time spent standing decreased during experiments. Licking the mouth was the most frequent behavior during transports, but not in the stationary car. In conclusion, a transport-induced stress response was evident in dogs. There was no habituation with repeated transports, and transported dogs may suffer from motion sickness.

**Abstract:**

This study aimed to characterize the response of transport-naïve dogs to one and two-hour road transports based on cortisol in saliva and blood plasma, heart rate, heart rate variability (HRV), neutrophil to lymphocyte (N/L) ratio and behavior. Two persons familiar to the dogs were present during transports and control experiments. We hypothesized that transport elicits a stress response, which decreases with repeated transports. Beagle dogs were allocated to three groups (*n* = 6 each). Group 1 served as control in the stable in week 1 and was transported for one hour in weeks 2, 3 and 4. Groups 2 and 3 served as controls in a non-moving vehicle and in the stable, respectively, in week 2. All three groups were transported for two hours in week 6. Cortisol concentration increased during transports (*p* < 0.001), and this increase remained constant with repeated transports. Cortisol release during two-hour transports was not affected by transport experience. Cortisol concentration increased twofold in plasma and eightfold in saliva, indicating an increase in free cortisol. The N/L ratio increased during transport (*p* < 0.05). Heart rate increased at the beginning of transport while HRV decreased (*p* < 0.001). Heart rate and HRV neither differed among weeks nor between animals with different transport experience. During transports, but also in the stationary vehicle, dogs were mostly sitting, and time spent standing decreased during experiments (*p* < 0.001). Licking the mouth was the most frequent behavior during transports but not in the stationary vehicle (*p* < 0.01). In conclusion, a transport-induced stress response was evident in dogs. There was no habituation with repeated transports, and transported dogs may suffer from motion sickness.

## 1. Introduction

Road transport is an artificial situation and a stressor for animals. Farm production animals, such as cattle, pigs and sheep, are shipped by road to slaughter, but also from breeding to fattening farms [1]. Horses are transported frequently to equestrian competitions, riding schools and for breeding purposes [2,3,4]. An abundance of information on stress and welfare during transport in horses [2,3,4,5,6,7], cattle [1,8], sheep and goats [9,10,11,12] and pigs [9,13] is available. In farm production animals, the potentially negative economic effects of transport, such as disease, mortality, injury, and carcass quality have also been addressed in scientific studies [1]. Based on changes in cortisol release, heart rate and heart rate variability, road transport is more stressful to horses than equestrian training or competitions [3,4,14,15,16,17]. 

Stress can be assessed by analysis of cortisol in saliva and blood plasma, heart rate and heart rate variability, changes in the white blood count and by observation of the animals´ behavior. Cortisol facilitates the response of an animal to stressful challenges through the modulation of energy metabolism and immune function [18]. Whereas plasma cortisol is mainly bound to carrier proteins, salivary cortisol mirrors unbound, i.e., free cortisol [19]. One effect of high cortisol concentration is neutrophilia, leading to an increase in the ratio of neutrophil granulocytes to lymphocytes [20].

Increases in heart rate indicate sympathetic activity of the autonomous nervous system. Heart rate variability, i.e., short-term fluctuations in heart rate, is essentially based on the antagonistic oscillatory influences of the sympathetic and parasympathetic nervous system on the nodus sinuatrialis of the heart. It, thus, reflects the prevailing balance of sympathetic and parasympathetic (vagal) tone. Heart rate variability is therefore used as an indicator for the response of the autonomic nervous system to stress. In general, decreases in the values of the HRV variables standard deviation of beat-to-beat (RR) interval (SDRR) and root mean square of successive RR differences (RMSSD) reflect a shift towards more sympathetic dominance, whereas increased values indicate a shift towards parasympathetic dominance [21]. 

Dogs frequently travel by car. They accompany their owners during vacations, weekends and sometimes to work, are transported to nearby parks or forests for a walk, to veterinary treatments, hunting events, dog shows and for breeding purposes. In comparison to production farm animals and horses, only limited information is available on the stress response of dogs to road transport. In a survey among dog owners, 24% of dogs were reported to have problems when travelling by car. When dogs had become used to travelling by car as puppies, the likelihood of transport-related problems at adult age was reduced, suggesting a certain degree of habituation [22]. In two experimental studies, road transport of dogs was associated with increases in heart rate, behavioral changes and an increase in the neutrophil to leukocyte ratio [23,24]. Changes in heart rate and behavior were less pronounced during slow cruising than normal driving [24].

The present study aimed at characterizing the stress response in transport-naïve dogs, based on physiological parameters and behavior, and at investigating whether dogs habituate to road transport when transported repeatedly. We hypothesized that dogs respond to road transport with a pronounced stress response, but this response decreases with repeated transports.

## 2. Materials and Methods

### 2.1. Animals

For the study, 18 Beagle dogs (females *n* = 4, castrated males *n* = 5, gonad-intact males *n* = 9) were available. Overall age of the animals was 2.7 ± 1.2 years (range 1.4–3.8 years) and weight was 14.0 ± 2.1 kg (range 10.5–18.4 kg, see Table 1).

Dogs were kept in groups of 2 to 4 animals in kennels and had access to an outdoor paddock of at least 20 m^2^ for 10 to 16 h per day. In addition, they were walked for one hour daily on the Vetmeduni Vienna Campus. Dogs were fed a commercial dog food once daily and water was available at all times. Dogs were either born and raised at Vetmeduni Vienna (*n* = 13) or brought to Vetmeduni Vienna from their breeding kennel four years ago at the age of four months (*n* = 5). Except for this, none of the dogs had been transported by car at any time.

### 2.2. Experimental Design

The study was approved by the competent authority for animal experimentation in Austria (Federal Ministry for Education, Science and Research, license number BMBWF-68.205/0150-V/3b/2019). 

Dogs were allocated to three different groups of 6 animals each. The age and sex of the animals was taken into account, in order to have equal numbers of male and female dogs in each group and no age difference between the groups (Table 1). Before the study, dogs were accustomed over four weeks to the transport cages, the heart rate recording equipment and to saliva sampling. The subsequent experiment lasted for six weeks. Group 1 served a control in the stable in week 1 and was transported for one hour at one-week intervals in weeks 2, 3 and 4 and for two hours in week 6. Group 2 served a control in the transports cage located in the non-moving transport vehicle in week 2 and was transported for two hours in week 6. Group 3 served as control in the stable in week 2 and was transported for two hours in week 6 (Table 2). Cardiac recordings, collection of saliva and blood and behavior observations were performed from one hour before, during and until two hours after transports and respective control phases. All experiments were performed between September and November of the same year. 

### 2.3. Transport

Dogs were transported in transport cages, approved by the International Air Transport Association (IATA) for Beagle-sized dogs (Vari Kennel Ultra, length 75 cm, width 50 cm, height 60 cm; Petmate, Arlington, TX, USA). Transport cages were placed on the floor of a minibus (Volkswagen Transporter, 2014 model, Volkswagen, Wolfsburg, Germany), with all rear seats removed. Between two and four dogs were transported together, but during transports and respective control phases in the stationary car, the animals had no visual contact with the other dogs in the car. Additionally, dogs could not see the outside environment during experimental phases in the car. Before the driving phase or control phase in the stationary car, dogs were allowed 10 min for familiarization with their cages in the shaded, non-moving car, followed by a 60 min (51 km; weeks 2–4) or 120 min (102 km; week 6) overland round drive at cruising speed. The route included approximately 25% city traffic and 75% four lane motorways. For the 2 h road transport in week 6, the same route was driven twice. The driver of the car and a second person accompanying the transports had handled the dogs during the four week accustomization phase before the study and were, thus, familiar to the dogs. The same two persons were also present during control experiments.

### 2.4. Heart Rate and Heart Rate Variability

Heart rate recordings were made with the Polar S 810i system (Polar, Kempele, Finland), as described previously for newborn foals [25], with the following modifications. The animal’s coat was clipped on the left side of the chest and the skin was cleaned with water. Ultrasound transmission gel (Gello Geltechnik, Ahaus, Germany) was applied liberally to ensure optimal conductivity. The Polar belt was fixed around the chest of the dogs with the transmitter placed in the clipped area. If necessary, belts were shorted in length. The dogs were fitted with a commercial dog body (VetMedCare, Koblach, Austria), covering the Polar belt and keeping it in the desired position.

The Polar system records cardiac beat-to-beat (RR) intervals. From the RR intervals, heart rate and heart rate variability (HRV) were calculated and analyzed with the Kubios HRV Software (Biomedical Signal Analysis Group, Department of Applied Physics, University of Kuopio, Finland) as described [25]. For HRV, the time domain parameter root mean square of successive beat-to-beat differences (RMSSD) was analyzed. Heart rate and HRV were averaged for 5 min intervals, starting at 60 min, 30 min, 15 min and 5 min before transport, 0 min, 5 min, 10 min, 15 min, 40 min and 60 min of one hour transports and additionally at 80 min, 100 min and 120 min of two-hour transports and at 5 min, 15 min, 30 min, 60 min, 90 min and 120 min after transports. Respective recordings were made during control experiments (cage in the stationary transport vehicle or stable).

### 2.5. Saliva and Blood Collection and Cortisol Analysis

Saliva for cortisol analysis was collected 60 min and 30 min and immediately before transports, at 20 min, 40 min and 60 min of one-hour transports, additionally at 90 min and 120 min of two-hour transports and 30 min, 60 min, 90 min and 120 min after transports. Respective samples were obtained during control experiments (cage in the stationary transport vehicle or stable). For the collection of saliva, a cotton roll (Salivette for cortisol, Sarstedt, Nümbrecht-Rommelsdorf, Germany) was grasped with a surgical clamp, gently inserted into the mouth of the dog, placed onto its tongue and left in place for 1 min, until it was well moistened. The salivettes were centrifuged at 1000× *g* for 10 min, and at least 1 mL of saliva was obtained and frozen at −20 °C until analysis. Blood samples for analysis of cortisol were collected by puncture of the saphenous vein and collected into heparinized tubes (Greiner Bio-One, Kremsmünster, Austria) 60 min before the start and 60 min after the end of the transports. Blood samples were centrifuged at 1000× *g* for 10 min and plasma was frozen at −20 °C until analysis. Respective saliva and blood samples were collected during both control experiments.

Concentrations of cortisol in saliva and blood were determined with an enzyme immunoassay without extraction (Demeditec Diagnostics, Kiel-Wellsee, Germany). Recovery of cortisol standard added to canine saliva and blood plasma was 95.8% and 96.1%, respectively. Serial dilution of canine saliva and blood plasma with assay buffer resulted in changes in optical density parallel to the respective standard curves. For saliva, the intra-assay coefficient of variation was 7.6%, the interassay coefficient of variation 3.3% and the minimal detectable concentration defined as two standard deviations from zero binding was 0.02 ng/mL. For blood plasma, the intra-assay coefficient of variation was 3.0%, the interassay coefficient of variation 10.3% and the minimal detectable concentration 0.35 ng/mL.

### 2.6. Hematology

Blood samples for hematology were collected as described for plasma cortisol into ethylene diamine tetra acetic acid (EDTA)-containing tubes (Greiner Bio-One, Kremsmünster, Austria) 60 min before the start and 60 min after the end of the two hour transports. Hematological parameters were determined with an automatic blood cell counter (V-Sight, Minerini Diagnostics, Vienna, Austria) with adapted veterinary software. Blood cell differential counts were checked by microscopy on Wright stained blood smears when scattergrams indicated problems with automated differentiation. For the study, the ratio of neutrophil granulocytes to lymphocytes (N/L ration) was analyzed.

### 2.7. Behavior Analysis

The behavior of dogs in the cage during all transports and the control experiment in the cage placed in the stationary car was recorded with a wide lens action camera (Apeman, GuangDong, China), fixed with a custom made holding device in front of each cage. All video recordings were analyzed by the same person using the Media Player computer program (Windows, Redmond, OR, USA). The video recordings were divided into 15 sec sequences, and behavior patterns were defined as described previously [26], with some modifications, as outlined in Table 3. For the posture of the dog (standing, sitting, sternal recumbency, lateral recumbency), only one category was possible at the same time, whereas for all other behaviors several options were possible.

### 2.8. Statistical Analysis

Statistical comparisons were made with the SPSS (Statistical Package for the Social Sciences) statistics program (version 26, IBM-SPSS, Armonk, NY, USA). Comparisons among groups 1 to 3 in weeks 2 and 6 were made by ANOVA with a general linear model (GLM) for repeated measures with time as within subject factor and group as between subject factor. For comparisons among different weeks in Group 1, the same test was used with both, time within each test and week as within subject factors. In the case of overall significant differences among groups, individual post hoc comparisons were made by the Tukey test. All values given are means ± SEM and a *p*-value <0.05 was considered significant.

## 3. Results

**Figure 1:**
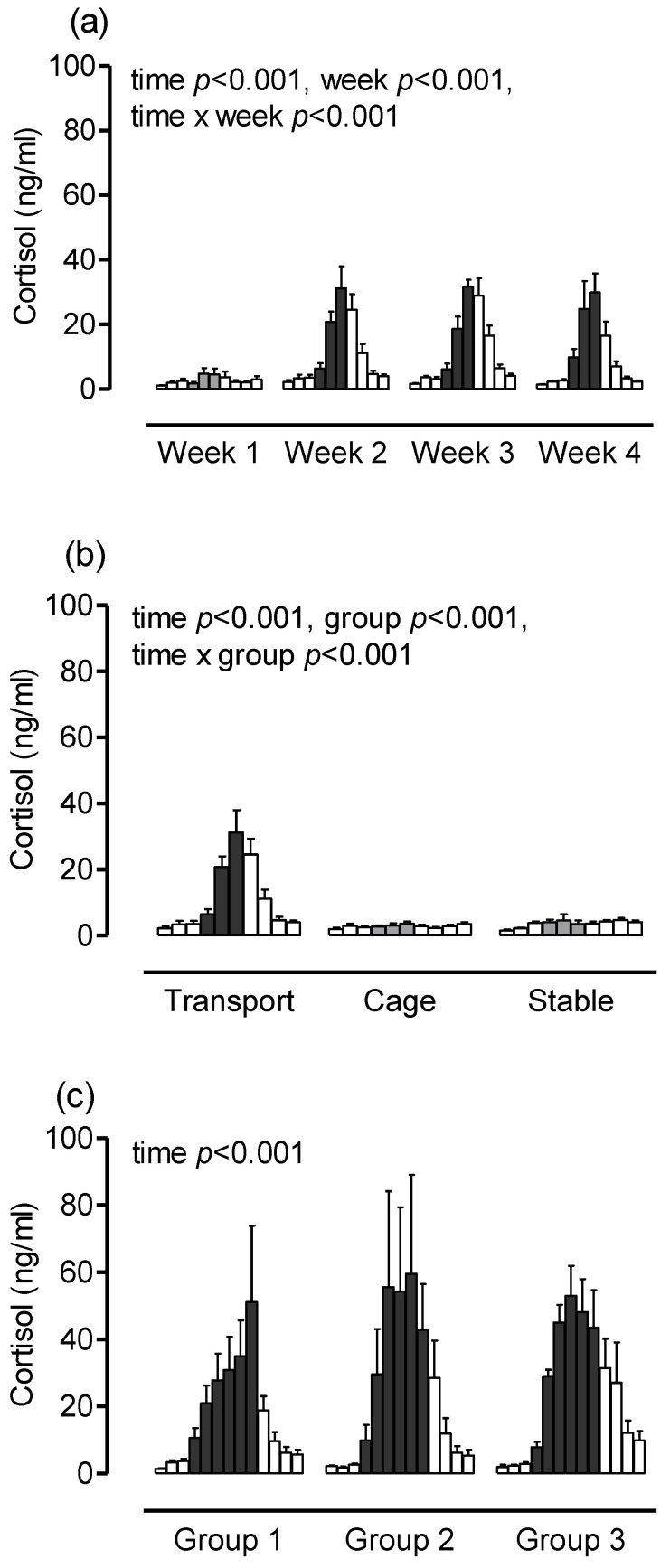
Concentration of cortisol in saliva (**a**) of the same dogs (*n* = 6), either resting in their stable (week 1), or transported by road for one hour at one week intervals (weeks 2–4); (**b**) three groups of dogs (n = 6 each), either transported by road for one hour, placed in the transport cage in a stationary car or remaining in their stable; and (**c**) the same three groups of dogs transported by road for two hours, open bars = 60 min pre and 120 min post-transport or control phase time, black bars = transport time and grey bars = respective control time, values are means ± SEM, significant differences are indicated in the figures.

Salivary cortisol concentration always increased when dogs were transported (*p* < 0.001; Figure 1a–c) and remained constantly low during control phases in the home stable (Figure 1a,b), as well as in the transport cage placed in the stationary transport vehicle (Figure 1b). When the same dogs (Group 1) were transported three times at one week intervals, cortisol release did not change with repeated transports (Figure 1a). Additionally, the cortisol release in response to a two hour transport in week 6 did not differ between groups with different transport experience (Figure 1c). Salivary cortisol concentration increased during the first hour of transport, and remained elevated during the second hour of transport, resulting in higher cortisol concentrations in the second compared to the first hour of transport (*p* < 0.001). There was, however, no significant difference between groups. Cortisol release into saliva calculated as area under the curve for the first and second hour of the two hour transport were 47 ± 12 and 105 ± 35, 69 ± 32 and 163 ± 74 and 61 ± 5 and 145 ± 24 ng/mL per min for groups 1, 2 and 3, respectively.

Cortisol in blood plasma was determined 60 min before and 60 min after two hour transports. In response to road transport, plasma cortisol concentration increased approximately twofold, while the corresponding increase in salivary cortisol concentration was approximately eightfold (Table 4).

**Figure 2:**
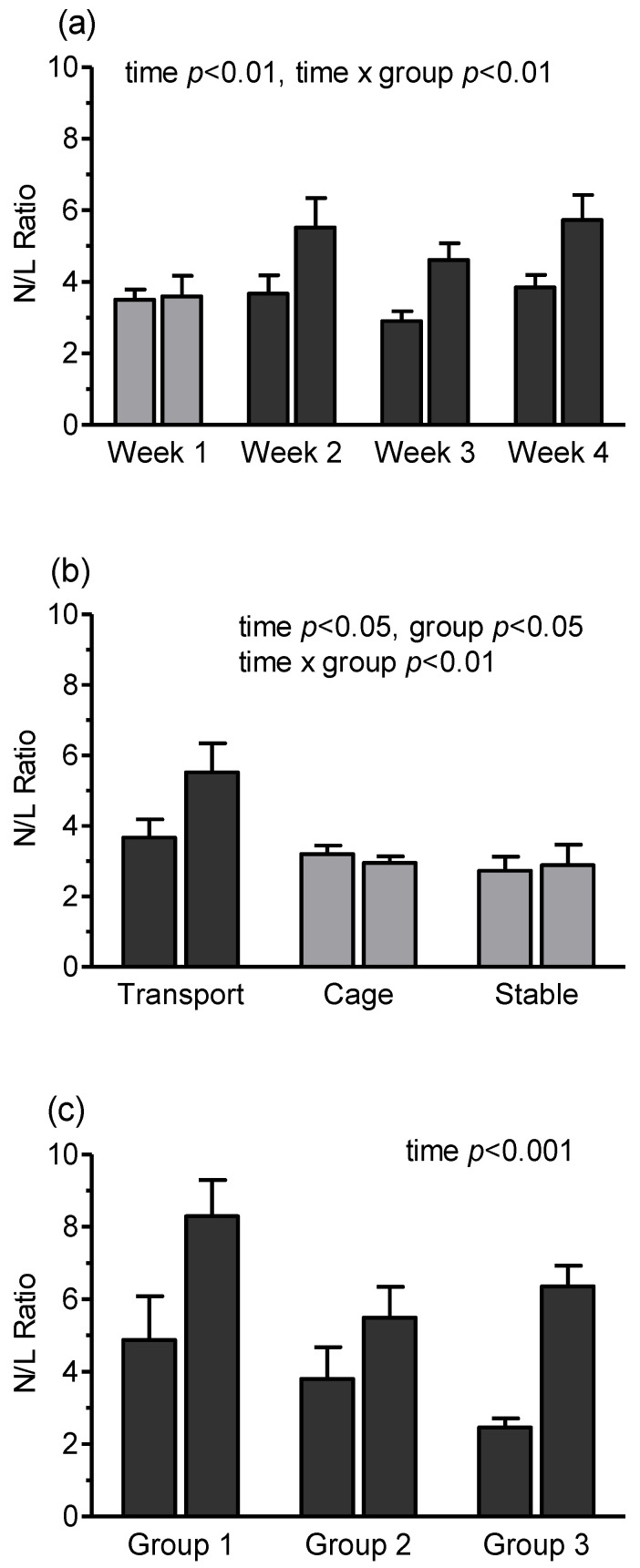
Neutrophil/lymphocyte (N/L) ratio at 60 min before and 60 min after experiments in (**a**) the same dogs (*n* = 6) either resting in their stable (week 1) or transported by road for one hour at one week intervals (weeks 2–4); (**b**) three groups of dogs (*n* = 6 each) either transported by road for one hour, placed in the transport cage in a stationary car or remaining in their stable; and (**c**) the same three groups of dogs transported by road for two hours, black bars = transport, grey bars = respective control experiments. Values are means ± SEM, significant differences are indicated in the figures.

Road transport induced an increase in the N/L ratio with a higher N/L ratio 60 min after the end of transport, compared to 60 min before transport (*p* < 0.05 to *p* < 0.001; Figure 2a–c). This increase did not change with repeated transports of the same dogs at one-week intervals (Figure 2a), was absent in dogs kept either in the transport cage in a stationary car or in their stable (Figure 2b) and did not differ among groups of dogs with different transport experience (Figure 2c). When the N/L ratio of Group 1 dogs was compared between week 2 (one-hour transport) and week 6 (two-hour transport), the increase was more pronounced in week 6 (from 4.9 ± 1.2 to 8.3 ± 1.0) than in week 2 (from 3.7 ± 0.5 to 5.5 ± 0.8; time *p* < 0.001, time x week *p* < 0.05). 

**Figure 3:**
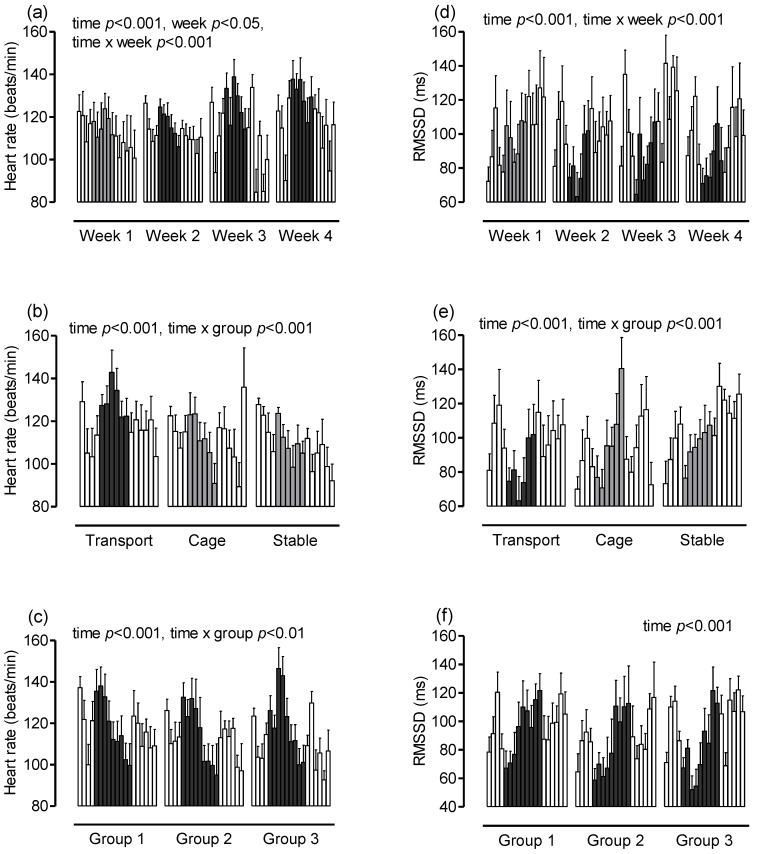
(**a**–**c**) Heart rate and (**d**–**f**) root mean square of successive beat-to-beat differences (RMSSD) in (**a**,**d**) the same dogs (*n* = 6) either resting in their stable (week 1) or transported by road for one hour at one week intervals (weeks 2–4); (**b**,**e**) three groups of dogs (*n* = 6 each) either transported by road for one hour, placed in the transport cage in a stationary car or remaining in their stable and (**c**,**f**) the same three groups of dogs transported by road for two hours, white bars = pre and post-transport or control phase time, black bars = transport time and grey bars = respective control time, values are means ± SEM, significant differences are indicated in the figures.

The average heart rate of dogs increased transiently at the beginning of all transports (*p* < 0.001; Figure 3a–c) but not during control experiments, neither when dogs were kept in the transport cages placed in the stationary vehicle, nor when the dogs remained in their stable during the control phase (week *p* < 0.05, time x week and time x group *p* < 0.001; Figure 3a,b). In dogs transported repeatedly at one week intervals, heart rate changes did not differ between transport weeks (Figure 3a). During the two hour transports, heart rate increased more in dogs that had neither been transported before nor spent a control phase in the transport cage in the non-moving transport vehicle (time x group *p* < 0.01; Figure 3c). 

The HRV variable RMSSD always decreased at the beginning of transports, and increased again during the second half of the transport phase (*p* < 0.001), but did neither differ significantly among transport weeks nor animal groups with different transport experience. During control experiments either in the stable or in a transport cage in the non-moving vehicle, changes in RMSSD were either absent or less pronounced than during transports (time x week and time x group *p* < 0.001; Figure 3d–f). In dogs transported repeatedly at one week intervals, heart rate changes did not differ among transport weeks (Figure 3d).

**Figure 4:**
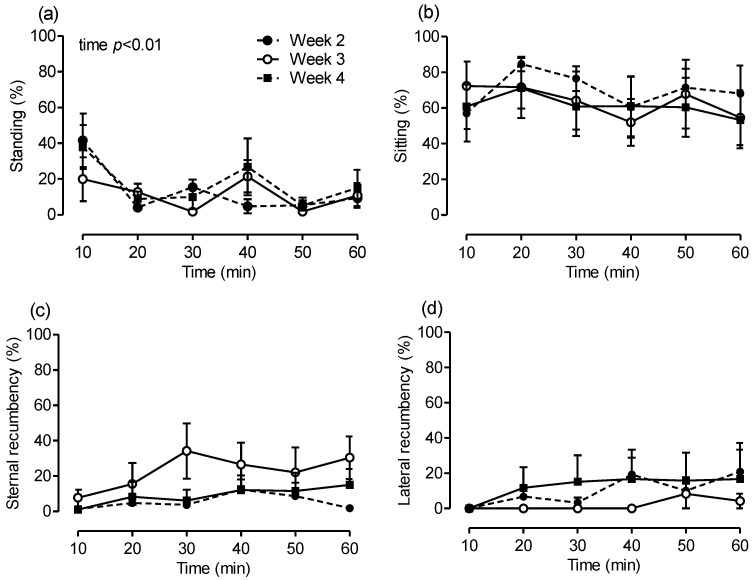
Time spent (**a**) standing, (**b**) sitting, (**c**) in sternal recumbency and (**d**) in lateral recumbency in dogs (*n* = 6) transported three times for one hour at one week intervals (weeks 2–4). Times are given as percentage for each position for subsequent 10 min intervals. Values are means ± SEM, significant differences are indicated in the figures.

**Figure 5:**
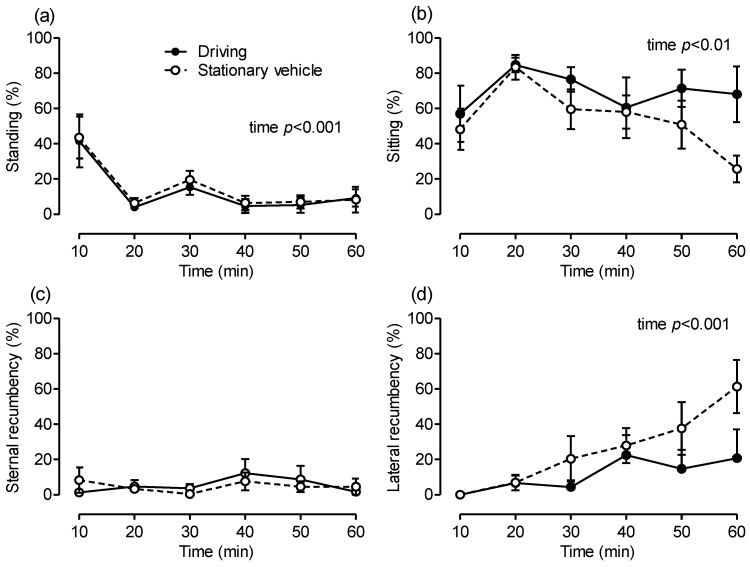
Time spent (**a**) standing, (**b**) sitting, (**c**) in sternal recumbency and (**d**) in lateral recumbency in dogs either transported for one hour (*n* = 6) or left in the transport cage in the stationary transport vehicle (*n* = 6; week 2). Times are given as percentage for each position for subsequent 10 min intervals. Values are means ± SEM, significant differences are indicated in the figures.

**Figure 6:**
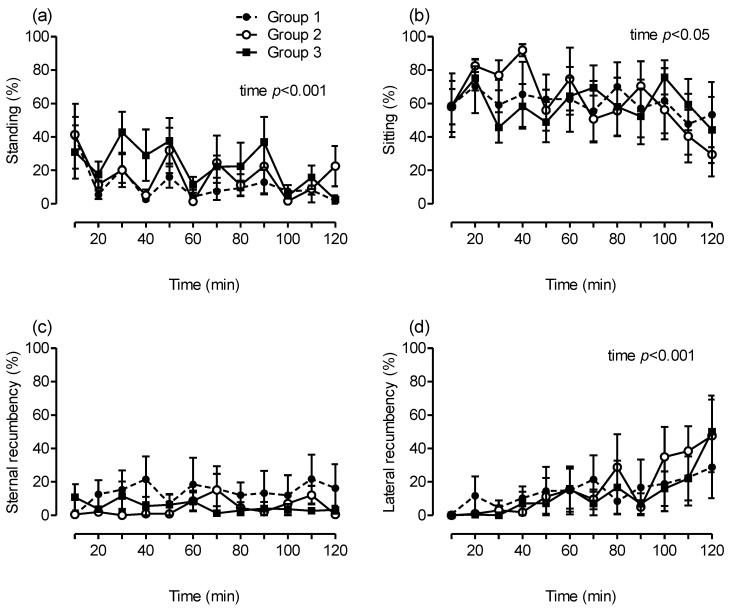
Time spent (**a**) standing, (**b**) sitting, (**c**) in sternal recumbency and (**d**) in lateral recumbency in dogs differing in transport experience transported for two hours (*n* = 6 per group). Times are given as percentage for each position for subsequent 10-min intervals. Values are means ± SEM, significant differences are indicated in the figures.

With regards to the posture adopted by the dogs in their transport cage either during transport or in the stationary vehicle, a sitting position predominated. Dogs transported for one hour three times at one week intervals, were sitting for more than half of the transport time. The percentage of time dogs were standing decreased within the first 10 min of transport (*p* < 0.001). The times dogs spent either standing, sitting or in sternal or lateral recumbency, did not change with repeated transports (Figure 4a–d). The dogs´ posture in the cage either in the moving or stationary vehicle did not differ significantly but, in both groups, the times spent standing and sitting decreased, while the time spent in lateral recumbency increased during the one hour transport and control phase (Figure 5a–d). There were similar changes during the two hour transports, with no significant differences among groups of dogs with different transport experience (Figure 6a–d). 

Out of 19 predefined behaviors, licking the mouth was the most frequently observed pattern during all transports, but not when dogs were confined to their cage in a stationary car (*p* < 0.01; Table 5). In dogs transported three times, digging-like movements were most evident on the first transport (*p* < 0.05), while the frequency of yawning increased with repeated transports (*p* < 0.05). During transports with a two hour duration, circling movements were more frequent (*p* < 0.01) in dogs that had not been transported before (Groups 2 and 3) than in the dogs that had been transported repeatedly during the experiment (Group 1). 

## 4. Discussion

A stress response to road transport was clearly evident in the dogs of our study. This conclusion is based on a marked increase in cortisol release, a shift towards a higher neutrophil to lymphocyte (N/L) ratio, an increase in heart rate and a decrease in the HRV variable RMSSD. In comparison to production farm animals and horses, only limited information is available on the stress response of dogs to road transport. Road transport of dogs in previous experiments was associated with similar increases in heart rate and the N/L ratio [23,24] but, to the best of our knowledge, there is no previous information on the stress response of transport-naïve dogs, nor their response to repeated road transport.

In transported dogs, the relative increase in cortisol concentration was more pronounced in saliva compared to blood. Salivary cortisol represents free and thus bioactive cortisol, while in blood, most cortisol is protein-bound and biologically non-active [19,27]. The more pronounced increase in salivary versus blood plasma cortisol concentration, thus, indicates that road transport of dogs not only increases total cortisol concentration in blood, but also induces the release of cortisol from its binding protein and, thus, an increase in the free cortisol fraction. In the present experiment, as in previous studies of our group on transport of horses [2,3,4], an increase in salivary cortisol concentration persisted throughout transports and values returned to the pre-transport baseline concentrations only after transport. Furthermore, salivary cortisol concentration was higher during the second compared to the first hour of a two hour transport, indicating continuous adrenocortical stimulation throughout the transport phase.

In our study, dogs were always transported late in the morning and control experiments were performed at the same time of the day. Effects of the diurnal rhythm in cortisol release with a peak in the morning followed by a gradual decline during the day into the night [26] on results of the study can be excluded. 

In contrast to persistently elevated cortisol concentration during transport, cardiac changes in transported dogs lasted only for approximately one hour or less. In contrast, in horses, an increase in heart rate persisted throughout one hour to eight hour transports, but heart rate returned to normal during longer road transports [2,3,4]. In pigs, loading onto the transport vehicle and the initial phase of transport were associated with an increase in heart rate, followed by a decrease and again an increase with prolonged transports [28]. The pattern of the stress response to transport thus differs among species, but within a species, it is also strongly determined by the previous experiences animals have had with vehicles and with people [29].

Heart rate variability of dogs determined as the time domain parameter RMSSD decreased transiently but returned to baseline values before the end of transport. Heart rate variability indicates fine tuning of the cardiac beat-to-beat interval by the autonomous nervous system, and a decrease in HRV is interpreted as a shift towards sympathetic dominance, while parasympathetic dominance leads to increased HRV [21]. In agreement with the changes in heart rate, the decrease in HRV, thus, suggests a transient sympathetic tone in dogs at the beginning of experimental transports. A stress response, however, was only evident when dogs were transported, and not when they were confined to the transport cage in the stationary vehicle. Transport was, thus, perceived as a stressful challenge, while mere confinement to the cage was not. 

Differences with regard to transport-induced changes in cortisol release, heart rate and HRV indicate that the sympathoadrenal and adrenocortical stress response is regulated, at least in part, independently from each other. Whereas, in healthy animals, an increase in cortisol release reliably indicates a stress response [18], changes in cardiac parameters do not necessarily indicate stress, but are also associated with physical activity or changes in emotional state. Although absolute HRV values differed between transport and control experiments, HRV decreased also transiently when dogs were either placed in a transport cage in a stationary vehicle or remained in their stable. We suggest that dogs experienced a state of arousal also during control experiments most likely in association with saliva sampling and the presence of humans. This interpretation is also supported by the complete lack of changes in salivary cortisol concentration and N/L ratio during both control experiments. 

An overall increase in the N/L ratio as in the present study has been demonstrated previously, not only in transported dogs [23], but also in cattle [8], pigs [13] and horses [30]. An increase in the N/L ratio is considered a cortisol-induced acute stress response of the immune system [31,32]. Lymphocytes exit the blood vessels and accumulate in peripheral tissues likely to be sites of infection, while neutrophils increase to eliminate pathogens that enter the blood stream [33]. 

Based on the physiological stress parameters cortisol, heart rate, HRV and N/L ratio, there was no habituation with repeated transports in the dogs of our study. This conclusion is based both on the dogs transported repeatedly for one hour at one-week intervals and on the two hour transports of three animal groups with different transport experience. It might be hypothesized that transport is perceived as less stressful when the dogs know its duration and destination from previous experience. In our study, this was not known during the initial transport, but during subsequent transports dogs may have learned that they always returned to their home kennels. The familiar kennels, however, represent a neutral environment. It would, thus, be of interest to study dogs transported to places associated with a strongly positive experience such as going to a forest or dog park to walk and play.

To the best of our knowledge, neither studies on road-transport of transport-naïve dogs nor on repeated road transport have previously been published. Unlike the dogs of the present experiment, transport-naïve three-year-old horses habituated rapidly to the condition. When transported repeatedly at one-week intervals, cortisol release became less pronounced with each transport, although a clear cortisol release was always evident, indicating a stress response even in transport-experienced horses [3]. Additionally, in steers, the transport-induced increase in the N/L ratio became less evident with repeated transports, suggesting habituation to repeated road transport also in bovines [34]. 

Being transported in a vehicle on the road is not a situation encountered by animals of any species in their respective natural environments. It is therefore not surprising to find a stress response to road transport in dogs at least in part comparable to what has been previously demonstrated in horses [2,3,4,35], sheep [9,10], cattle [1,8] and pigs [9,13]. On the other hand, dogs in our study spent most time in a sitting or recumbent posture while horses and cattle are usually standing. Standing animals must constantly balance turns, accelerations and sudden braking of the moving vehicle [1,4,5,10], which is less challenging in a sitting and much smaller animal. Therefore, transport may be a less stressful situation in dogs than in horses.

Behavior analysis revealed less effects of road transports than physiological stress parameters. When placed in the transport cage, either in the moving or in the stationary vehicle, most dogs were in a sitting position. Throughout the one and two hour transports, but also during the control phase in the cage, the time dogs were standing decreased, and the time spent in lateral recumbency increased. These changes in position were, thus, not induced by the transport. We suggest that the dogs responded to a new situation, i.e., to being placed in the transport cage with a certain curiosity. They were thus standing and expecting the investigator to interact with them. When the situation remained the same, they finally laid down and waited. It is also possible that the animals initially expected to be released again and after some time accepted that they had to stay in the transport cage.

Out of the behavior patterns analyzed in the stationary and the moving car, the time dogs spent licking their mouth differed between the two situations and was more evident in the moving car. Mouth licking was also the most frequent behavior seen during the two hour transports, and did not differ between the three groups of dogs with and without previous transport experience. With repeated transports the time spent with digging-like movements decreased while yawning increased. Furthermore, dogs with transport experience showed fewer circling movements during the two hour transport than dogs without transport experience. We suggest that the behavioral changes in dogs of the present study in response to transport to some extent are caused by motion sickness. Motion sickness has been suggested to be triggered by difficulties which arise in the programming of movements of the eyes or head when there is a mismatch between actual versus expected sensory inputs. Its presentation includes the gastrointestinal tract, central nervous system and autonomic symptoms [36]. Symptoms compatible with motion sickness have been described in sheep transported by ship [33] and in pigs during road transport [9]. Behaviors indicating travel sickness in pigs included repetitive chewing, foaming at the mouth and bouts of sniffing the air [9]. Licking their mouth and yawning in transported dogs of the present study might be a comparable sign of sickness. Travel sickness at least in some dogs will occur regularly when they are travelling by car, whereas the stress response to transport as in other animal species can be expected to decrease over time.

Our present results do not exclude that a certain habituation of dogs to road transports occurs over longer time periods, i.e., months or even years. Results of a questionnaire study among dog owners suggest that dogs used to travelling by car since puppy age were less likely to respond negatively to road transport than dogs transported as adults only. According to their owners’ perception, however, 24% out of close to 1000 dogs experienced problems when transported by car [22]. This suggests that although a certain degree of adaptation occurs in most dogs over longer times, transport is still perceived as stressful or negative by a substantial percentage of dogs. 

During transports, dogs in our study were accompanied by two familiar persons who were in daily contact with the animals during the four weeks before the experiment started. Although the interaction between these persons and the experimental dogs had been less intense than between typical family dogs and their owners, a certain reassuring effect of these persons during transports can be assumed as has been described for dogs exposed to a threatening stranger either with or without the dogs´ owners being present [37]. A total lack of familiar people would most probably further increase the animals´ stress response to road transport.

## 5. Conclusions

Transport-naïve dogs perceive road transport as a stressful challenge. They do not adapt to repeated transports within a few weeks although habituation over a longer time-period cannot be excluded. In addition to perceiving transport as a stressful challenge, dogs may suffer from motion sickness in a moving vehicle, which prevents a more rapid habituation. 

## Figures and Tables

**Table 1:** Age, weight and sex of dogs in the different experimental groups.

	Group 1	Group 2	Group 3
Age (years)	2.4 ± 1.4	2.8 ± 1.2	2.9 ± 1.3
Weight (kg)	13.8 ± 2.5	14.8 ± 1.7	13.5 ± 2.0
Female/intact male/castrated male	2/3/1	1/3/2	1/3/2

No significant differences among groups.

**Table 2:** Experimental groups and study design.

Week	Group 1 (*n* = 6)	Group 2 (*n* = 6)	Group 3 (*n* = 6)
1	Control phase in stable (1 h)	--	--
2	Road transport (1 h)	Control phase in cage (1 h)	Control phase in stable (1 h)
3	Road transport (1 h)	--	--
4	Road transport (1 h)	--	--
5	--	--	--
6	Road transport (2 h)	Road transport (2 h)	Road transport (2 h)

**Table 3:** Behavioral patterns analyzed during transport and control phases in the stationary vehicle.

standing	standing with all four paws but no other parts of the body in contact with the ground
sitting	front legs stretched and hindlegs flexed with the hindquarters in contact with the ground
sternal recumbency	lying on chest and belly, with the head up
lateral recumbency	lying on the right or left side of the body
autogrooming	licking- and biting self, behavior directed towards the subject’s own body
digging	scratching the floor with the forepaws in a way that is similar to when dogs are digging holes
circling	continuous walking in short circles
whimpering	low, intermittently crying
yowling	long, loud crying
licking mouth	licking around the mouth with the tongue
yawning	involuntary wide opening of the mouth together with a long and deep inhalation, eyes usually closed
panting	an increased frequency of inhalation and exhalation often in combination with the opening of the mouth
undressing	attempt to take off the body by scratching with their paws and/or tearing with the teeth
cage licking/nibbling	the cage is licked with the tongue/nibbled with the incisors
body shaking	vigorously oscillating the head and body on its longitudinal axis
wallowing	turning over to the back
nosing	the nose is moved along objects and/or clear sniffing movements are exhibited
trembling	a clear shivering of the body
vomiting	involuntary, forceful expulsion of the stomach contents through the mouth
tail put between legs	the tail curled forward between the hind legs
tail wagging	repetitive wagging movements of the tail
tilting over	falling over e.g., after braking of the car
urinating	sudden release of urine from the urinary bladder through the urethra to the outside of the body

**Table 4:** Cortisol in saliva and blood plasma of dogs of all three groups combined (*n* = 18) before and after a two hour road transport and salivary cortisol concentration expressed as percentage of blood plasma cortisol concentration, values are means ± SEM.

	Saliva	Blood Plasma	Percent
Before transport (−60)	1.9 ± 1.0 ng/mL	33.6 ± 16.2 ng/mL	5.7%
After transport (+60)	16.5 ± 19.4 ng/mL	60.1 ± 42.4 ng/mL	27.5%
	*p* < 0.01	*p* < 0.05	*p* < 0.01

**Table 5:** Occurrence of behavior patterns during one hour road transport or in control dogs in the stationary car (data indicate number out of 240 intervals of 15 s each in which the behavior was shown) and during two hour road transports (data indicate number out of 480 intervals of 15 s each, in which the behavior was shown), *n* = 6 per group, data are mean ± SEM.

Behavior	Week 2 1 h Transport (Group 1)	Week 3 1 h Transport (Group 1)	Week 4 1 h Transport (Group 1)	Sign. Weeks 2–4	Week 2 Cage (Group 2)	Sign. Groups 1 vs. 2, Week 2	Week 6 2 h Transport (Group 1)	Week 6 2 h Transport (group 2)	Week 6 2 h Transport (Group 3)	Sign. Groups 1–3, Week 6
autogrooming	3.5 ± 1,5	1.3 ± 0.7	0.8 ± 0.3		9.2 ± 3.9		8.8 ± 5.6	1.0 ± 0.5	2.0 ± 0.6	
digging	9.5 ± 3.7	0.7 ± 0.7	0.3 ± 0.2	*p* < 0.05	4.5 ± 1.9		1.0 ± 0.7	6.6 ± 3.6	11.5 ± 6.1	
circling	13.5 ± 1.4	9.8 ± 3.5	9.2 ± 3.2		11.8 ± 6.8		9.0 ± 2.5	30.4 ± 5.5	38.8 ± 7.5	*p* < 0.01
whimpering	5.0 ± 3.9	0	3.0 ± 1.9		5.0 ± 2.7		0	2.8 ± 2.8	0.2 ± 0.2	
yowling	1.3 ± 1.1	0	0		1.0 ± 0.5		0	0.4 ± 0,4	0	
licking mouth	40.7 ± 13.3	68.3 ± 24.6	54.3 ± 16.9		1.5 ± 1.5	*p* < 0.01	67.8 ± 28.2	61.4 ± 16.2	61.3 ± 14.6	
yawning	2.0 ± 1.1	7.3 ± 2.0	6.3 ± 1.9	*p* < 0.05	0.5 ± 0.5		10.8 ± 3.2	11.0 ± 4.2	3.7 ± 1.9	
panting	11.8 ± 11.8	12.0 ± 12.0	9.5 ± 9.5		0.8 ± 0.8		4.7 ± 4.5	59.6 ± 59.6	64.2 ± 44.3	
undressing	0	0	0		5.3 ± 4.4		0	1.2 ± 1.2	0.3	
cage licking/nibbling	2.0 ± 1.0	0.2 ± 0.2	0.8 ± 0.3		8.3 ± 7.5		0.5 ± 0.2	3.6 ± 2.7	6.8 ± 4.5	
body shaking	0	0.3 ± 0.5	0.2 ± 0.2		1.0 ± 0.8		1.0 ± 0.7	1.6 ± 0.9	1.7 ± 1.7	
wallowing	1.2 ± 1.2	0	0		0.5 ± 0.5		0.2 ± 0.2	0	1.5 ± 1.1	
nosing	0.5 ± 0.3	24.3 ± 7.0	0	*p* < 0.01	0.8 ± 0.8		0	2.6 ± 1.8	0	
trembling	5.0 ± 3.2	17.2 ± 5.6	2.8 ± 1.6	*p* < 0.05	0		3.7 ± 1.9	1.4 ± 1.4	5.0 ± 2.9	
vomiting	0.5 ± 0.3	0.3 ± 0.3	0.3 ± 0.2		0		1.5 ± 1.1	0.6 ± 0.4	1.0 ± 0.7	
tail put between legs	3.2 ± 7.8	0	0		0		5.8 ± 5.8	0	0	
tail wagging	0	0	1.0 ± 0.8		0		1.0 ± 0.5	0.8 ± 0.8	10.2 ± 9.4	
tilting over	0	0	0		0		1.7 ± 1.7	0	1.5 ± 1.1	
urinating	0	0	0		0		0	0	0.7 ± 0.7	

Sign: significant differences among weeks (repeated transports in Group 1) or groups, grey shading indicates values differing among groups or within Group 1 among different weeks.
